# Morphology-Based Prediction of Osteogenic Differentiation Potential of Human Mesenchymal Stem Cells

**DOI:** 10.1371/journal.pone.0055082

**Published:** 2013-02-21

**Authors:** Fumiko Matsuoka, Ichiro Takeuchi, Hideki Agata, Hideaki Kagami, Hirofumi Shiono, Yasujiro Kiyota, Hiroyuki Honda, Ryuji Kato

**Affiliations:** 1 Department of Biotechnology, Graduate School of Engineering, Nagoya University, Nagoya, Aichi, Japan; 2 Department of Engineering, Nagoya Institute of Technology, Nagoya, Aichi, Japan; 3 Tissue Engineering Research Group, Division of Molecular Therapy, The Institute of Medical Science The University of Tokyo, Tokyo, Japan; 4 Department of Oral and Maxillofacial Surgery, Matsumoto Dental University School of Dentistry, Shiojiri, Nagano, Japan; 5 Nikon Corporation, Tokyo, Japan; 6 Department of Basic Medicinal Sciences, Graduate School of Pharmaceutical Sciences, Nagoya University, Nagoya, Aichi, Japan; Foundation for Applied Medical Research, Spain

## Abstract

Human bone marrow mesenchymal stem cells (hBMSCs) are widely used cell source for clinical bone regeneration. Achieving the greatest therapeutic effect is dependent on the osteogenic differentiation potential of the stem cells to be implanted. However, there are still no practical methods to characterize such potential non-invasively or previously. Monitoring cellular morphology is a practical and non-invasive approach for evaluating osteogenic potential. Unfortunately, such image-based approaches had been historically qualitative and requiring experienced interpretation. By combining the non-invasive attributes of microscopy with the latest technology allowing higher throughput and quantitative imaging metrics, we studied the applicability of morphometric features to quantitatively predict cellular osteogenic potential. We applied computational machine learning, combining cell morphology features with their corresponding biochemical osteogenic assay results, to develop prediction model of osteogenic differentiation. Using a dataset of 9,990 images automatically acquired by BioStation CT during osteogenic differentiation culture of hBMSCs, 666 morphometric features were extracted as parameters. Two commonly used osteogenic markers, alkaline phosphatase (ALP) activity and calcium deposition were measured experimentally, and used as the true biological differentiation status to validate the prediction accuracy. Using time-course morphological features throughout differentiation culture, the prediction results highly correlated with the experimentally defined differentiation marker values (R>0.89 for both marker predictions). The clinical applicability of our morphology-based prediction was further examined with two scenarios: one using only historical cell images and the other using both historical images together with the patient's own cell images to predict a new patient's cellular potential. The prediction accuracy was found to be greatly enhanced by incorporation of patients' own cell features in the modeling, indicating the practical strategy for clinical usage. Consequently, our results provide strong evidence for the feasibility of using a quantitative time series of phase-contrast cellular morphology for non-invasive cell quality prediction in regenerative medicine.

## Introduction

Mesenchymal stem cells (MSCs) are a useful cell source for tissue engineering and regenerative medicine of various tissues because of their multi-lineage differentiation capacity (e.g., osteogenic, chondrogenic, adipogenic, neurogenic, and myogenic) [1]–[3]. Although MSCs can be harvested from various tissues, including adipose tissue and dental pulp, bone marrow derived MSCs (BMSCs) have a well-described *in vivo* bone-forming capacity and are widely used for clinical bone regenerative therapies [4]–[6]. Several groups, including ours, have been successful in clinical bone tissue engineering using human bone marrow mesenchymal stem cells (hBMSCs) [7]–[9]. In spite of documented clinical successes of bone regeneration with hBMSCs, robust therapeutic efficacy able to withstand the large variation among patients remains a challenge. Therefore, practical and effective cell-quality assurance methods are a necessary approach to address the unmet need of minimizing variability in patient outcomes.

Previous works aimed at characterizing BMSC osteogenic potential have mainly focused on methods that damage cells (e.g., staining, gene expression, etc.) [10]. These conventional techniques limit clinical translation in two ways. First, the destructive nature of the measurements consumes cellular material that would otherwise be useful for therapy. Second, the sample measurements are terminal endpoints, in part due to the irreversible damage incurred by the cells from the measurement procedure. As a result, repeated measurement on the same cellular sample is not possible and longitudinal sampling consumes more material.

Currently, the daily monitoring of cellular morphology by microscopy is combined with minimum sampling for biochemical markers to serve as the routine cellular quality assessment during the expansion culture process. Qualitative microscopic examination and the consumptive nature of the biochemical assays impose a limit on the predictive control currently available in clinical practice. A quantitative, non-invasive method for predicting cellular osteogenic potential and quality is needed to better anticipate clinical outcomes.

Cellular morphology has historically been used as an important indicator to characterize present and assess cell quality. Several reports describe correlations between osteogenic differentiation potential and cellular morphology. Kelly *et* *al*. have reported that cell geometry is highly correlated with differentiation into osteogenic lineages [11]. Takagi *et* *al*. have also reported that the cell roundness of hBMSCs is highly correlated with the expression of osteogenic differentiation marker genes [12]. In addition to the above examples that match morphology and cell potential, there are increasing numbers of reports describing image-based cell assessment methods. The popularity of fluorescence-labeled imaging methods in high content cellular screening has outpaced methods with non-labeled image-based assessment; however these approaches retain some technical drawbacks, which do not necessarily improve upon the non-labeled methods [13]–[16].

In this study, we aimed to demonstrate the efficacy of the non-invasive prediction model, which only uses cellular morphology features to forecast the osteogenic differentiation potential of hBMSCs. Specifically, the outcomes of two biochemical osteogenic markers were quantitatively forecast by two types of prediction models: (1) the alkaline phosphatase (ALP) activity 14 days after differentiation, designated as “D14_ALP model”, and (2) the calcium deposition rate 21 days after differentiation, designated as “D21_Ca model”. ALP activity is a BMSC differentiation marker; however measuring ALP activity alone is not sufficient for predicting *in vivo* bone formation. Compared to ALP, calcium deposition rate is an osteogenic differentiation marker that highly correlates with *in vivo* bone formation. However, since calcium deposition is a late phenotypic marker, which appears beyond the optimal implantation stage, it is not commonly applied as a clinically useful marker. Overall, it is impossible to measure both markers with the same cell sample or quantitatively predict the measurement results using conventional methods.

To advance this field, we aimed to investigate whether a morphology-based prediction model is capable of quantitatively predicting both ALP activity and calcium deposition rate. Further on, to demonstrate the clinical feasibility of our resulting morphology-based prediction models we examined practical considerations for use in the clinic in order to predict osteogenic potential for new patients scheduled for cell therapy. Two simulation scenarios were carefully examined: (Scenario I) Prediction of osteogenic differentiation potential of a new patient's cells by a model trained with historical patient data; (Scenario II) Prediction of osteogenic differentiation potential of a new patient's cells by a combination of historical patient data and partial culture imaging data from the early stage expansion of the new patient's own cells.

## Results

### Biological/morphological changes during osteogenic differentiation culture

**Figure 1:**
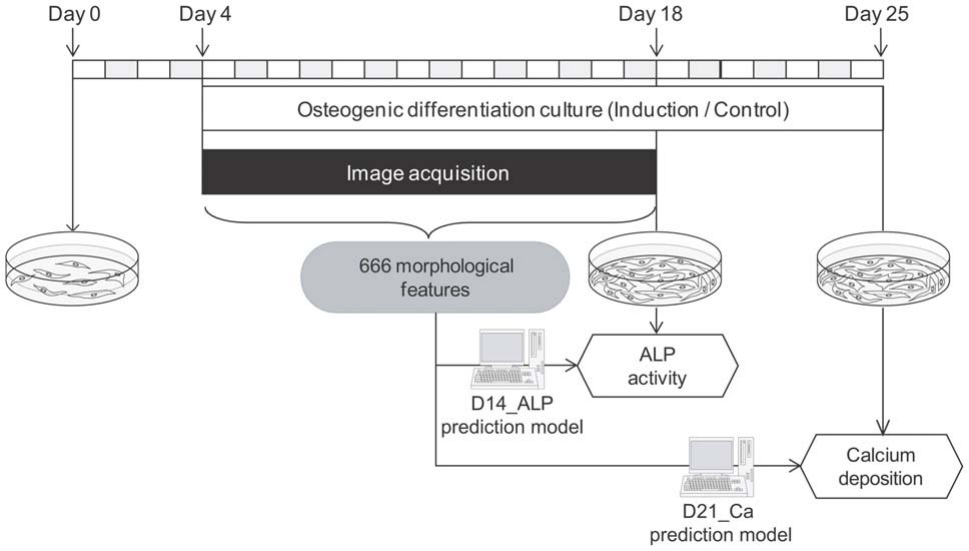
Schematic illustration of the experimental scheme for the prediction of osteogenic differentiation potential using multiple and time-course morphological features. hBMSCs were cultured in non-induction medium in first 4 days, then the medium was replaced with osteogenic induction medium only for the Induction sample. From day 0 to day 14, cell images were automatically acquired by BioStation CT every 8 hours. ALP activity and calcium deposition rates were evaluated on days 14 and 21, respectively. Using multiple morphological features covering 2 weeks culture, two types of hBMSC osteogenic differentiation evaluation results were predicted by individual prediction models.

hBMSCs were either cultured in differentiation induction medium or in non-induction medium according to the protocol illustrated in Fig. 1, which was based on the clinical jaw bone therapy protocol used by our group [7]. Three lots of cells, passaged three times per lot, were used to assess “patient-derived variance” and “processing-derived variance.” After the image acquisition period during differentiation culture, ALP activity was measured from the same well that the images were acquired. After an additional week of differentiation culture, calcium deposition rate was quantified.

**Figure 2:**
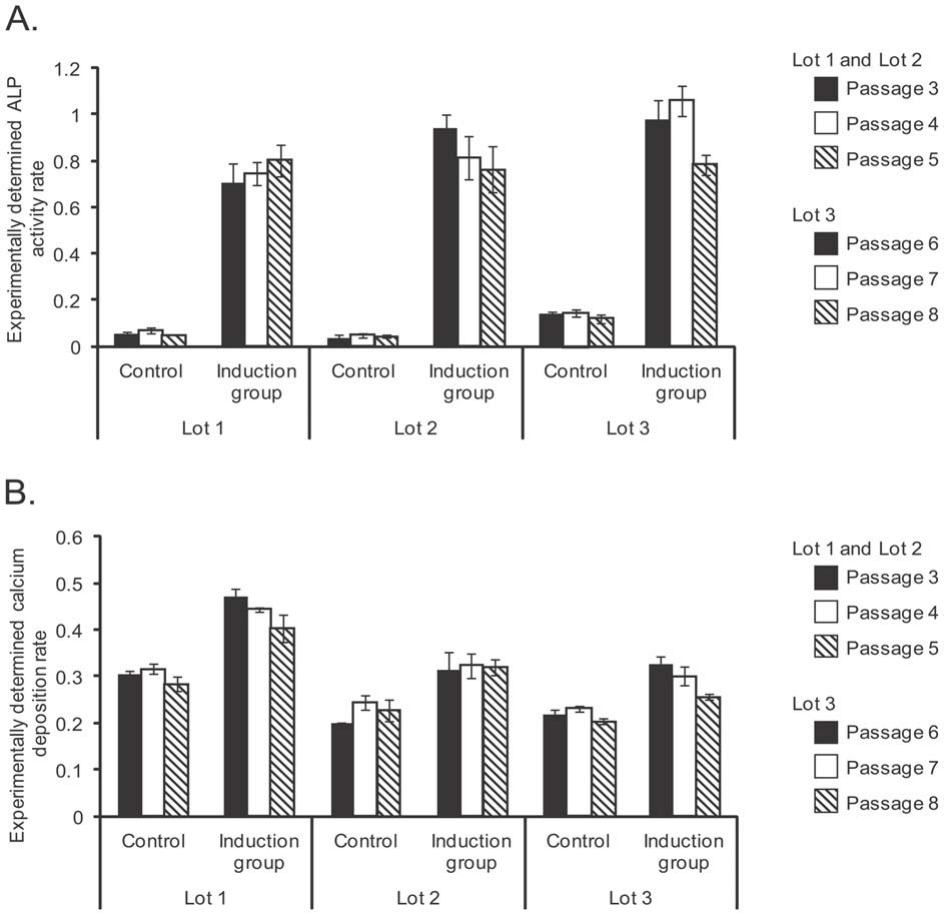
Experimentally determined biological results after the osteogenic differentiation. A: Experimentally determined ALP activity rate on day 14 of differentiation. B: Experimentally determined calcium deposition rate on day 21 of differentiation.

All cell lots at all passages in the induction groups showed a clear increase in ALP activity compared to the control groups (Fig. 2–A). The calcium deposition rate was also significantly higher in the induction group than the control group among all lots and all passages (Fig. 2–B). However, greater variation was observed in the calcium deposition assays compared to the ALP assays. This result reflects the fact that ALP activity measurements add information of osteognic differentiation, but does not qualify as a marker of further osteogenic maturation potential even in *in vitro*.

**Figure 3:**
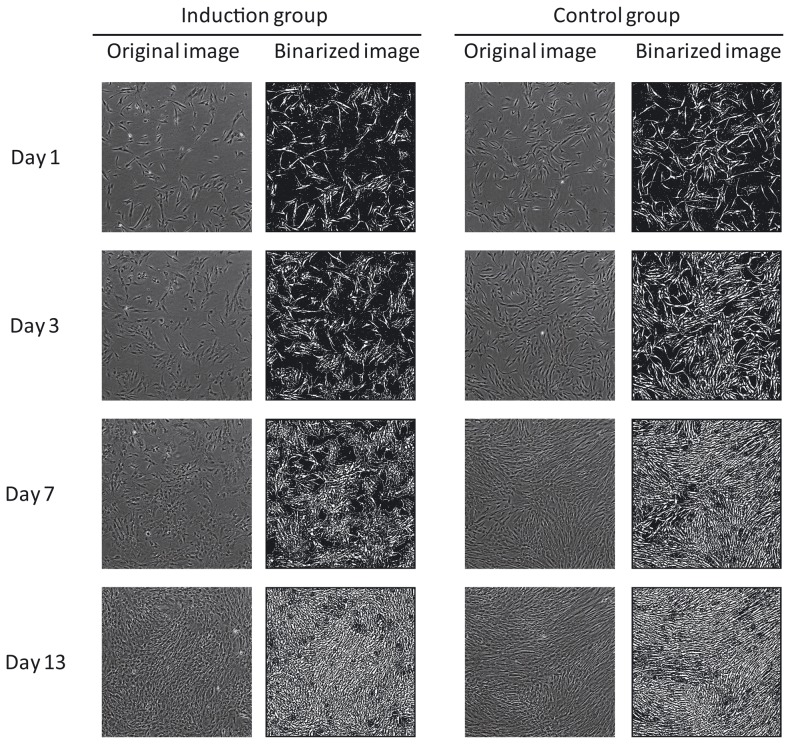
Phase contrast raw image from BioStation CT and its processed image. The images of beginning (day 1), middle (day 3 and 7), and the end (day 13) in the induction period of Lot 1 are indicated as examples. Raw images were binarized with MetaMorph.

**Figure 4:**
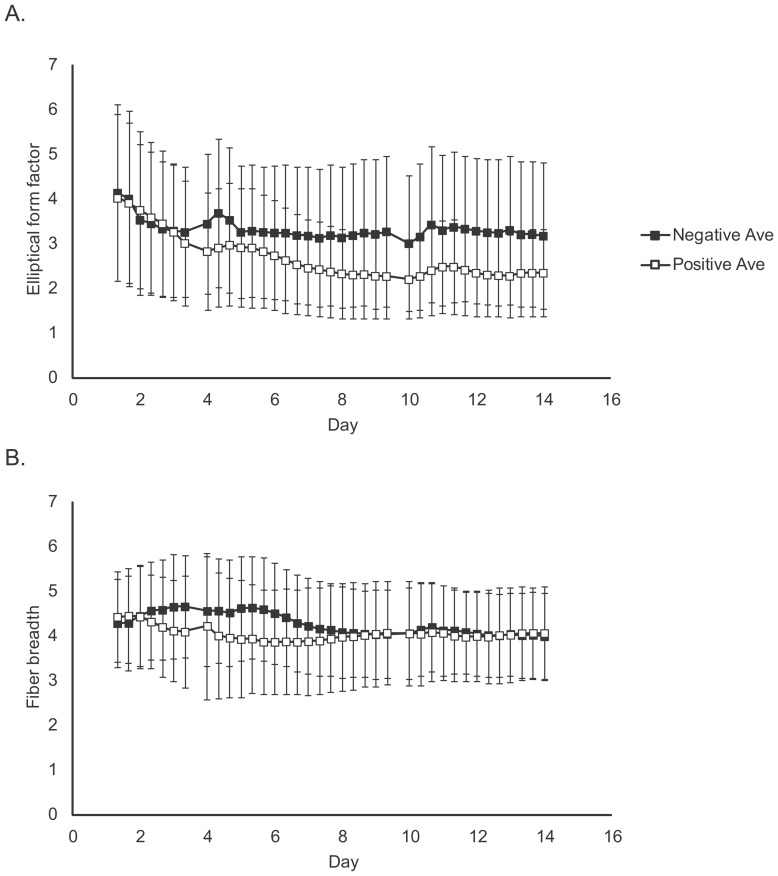
Time series changes of characteristic morphological features. From the 9 morphological features measured, elliptical form factor (A) and fiber breadth (B) of Lot 1 are indicated as representative examples. The symbols indicate the mean value of each morphological feature from all cells in one condition (3 wells ×5 view fields). Roughly, 4,000 to 40,000 cells were measured for the mean. Standard deviations are shown as error bars.

In contrast to biochemical measurements, which exhibited a noticeable pattern after several weeks of culture, a signature pattern using morphological measurements was found within 7 days of differentiation culture (Fig. 3). For all cell lots at the 7 day time point, cell morphology in the induction group was observed as flat and spread in multiple two dimensional directions, as compared to the fibroblast-like sharp spindle shape of the control group. By summarizing the quantitative morphological changes in all cells under the same culture conditions through image analysis, an early indication of the cellular phenotype was apparent. For some morphological features, such as Elliptical form factor (the ratio of the object's width to its length) or Fiber breadth (the width of an object modified as a straight fiber), a statistically significant difference (p<0.01) between induction and control groups could be identified at a very early culture stage (Fig. 4). Elliptical form factor of Lot 1 was significantly different (P<0.001) from day zero at day three of differentiation culture and then throughout the differentiation period. Although these types of morphological differences suggest a relationship to osteogenic induction, they are insufficient to quantitatively predict the final cellular state. To improve predictive power, a machine learning approach was taken to construct a computational model for quantitative prediction and determine the best combination of morphological features to use.

### Prediction of osteogenic differentiation potential using multiple and time-course morphological features

Standard practice for bone regeneration therapy is to start by expanding a new patient's cell material to a certain yield, then applies an osteogenic differentiation protocol up until the day of therapy. Variations in the quality of a new patient's starting material can be exacerbated by the stresses of cellular expansion. For these reasons a model for characterizing the regenerative capacity of a patient's cell source, including quality, yield and most importantly osteogenic potential, would add tremendous value to current standard practice.

**Figure 5:**
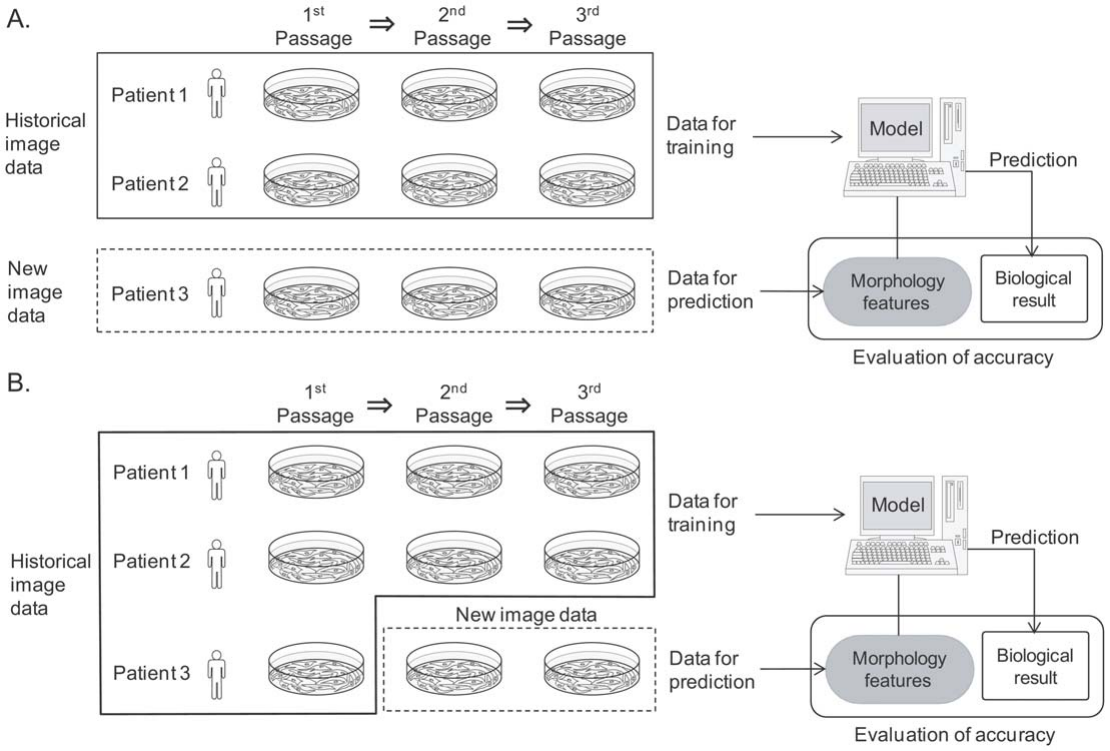
Schematic illustration of two scenarios examined to simulate clinical feasibility. A: (Scenario I) New patient prediction scheme: Trained by historical patient dataset only. Images from all passages of patient 3 were used for prediction. B: (Scenario II) Ongoing patient prediction scheme: Trained by historical patient datasets and a partial dataset from the new patient. For example, for the prediction of cell potential of patient 3, Scheme I uses images of patient 1 and 2 only. Scheme II used images of patient 1 and 2, together with some images from patient 3.

Two scenarios were designed to simulate anticipated clinical situations available for applying morphology-based prediction models to assess new patient cellular quality. Scenario I: Prediction of new patient BMSC osteogenic potential using a model trained with historical patient data (Fig. 5–A). Scenario II: Prediction of new patient BMSC osteogenic potential using a model trained with historical patient data in addition to data derived from the new patient material (Fig. 5–B). The accuracies of both D14_ALP and D21_Ca models were evaluated in each scenario. Nine morphological features were evaluated from 37 time points over 14 days and complied from 666 image-based input features. The corresponding biochemical differentiation markers from each of the 54 samples were also evaluated. We selected Ridge regression as the machine learning modeling method for linking morphological features to the biomarker measurement results [17]. This method was chosen since Ridge regression is a type of standard regression model that eliminates the multicolinearity problem in multivariate models [18].

#### Scenario I: New patient prediction scheme

The new patient prediction scheme is designed to simulate the clinical situation where evaluating a new patient's cell quality can be accomplished quickly and reliably (Fig. 5–A). With this scheme, the prediction model can be prepared previously by historical image data from other patients. This model aspires to require no previous data from the new patient.

**Figure 6:**
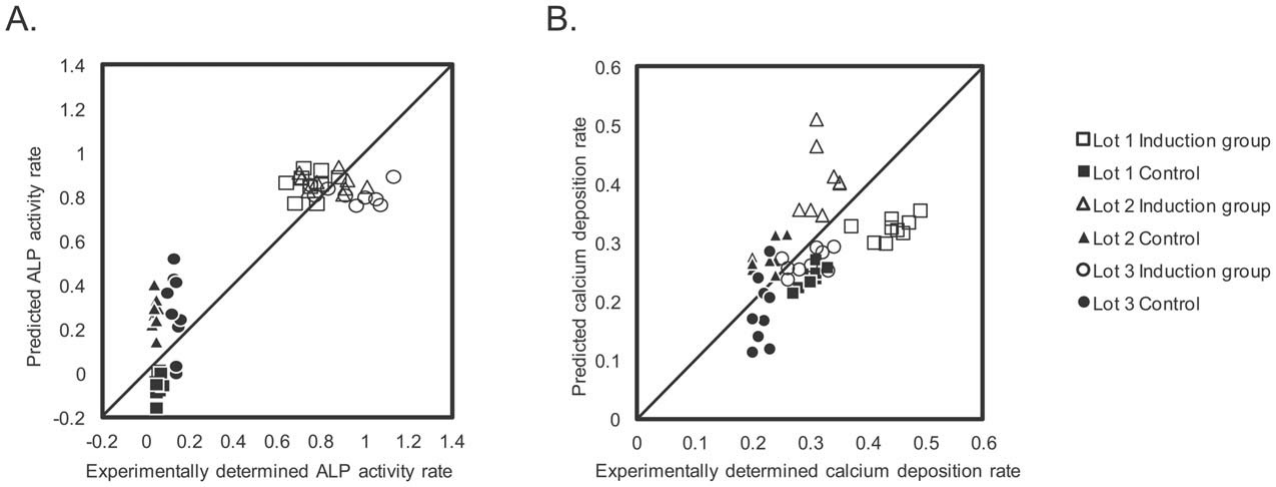
Prediction accuracies in the new patient scheme. A: Scatter plot of experimentally determined values versus predicted values in D14_ALP model, B: Scatter plot of experimentally determined values versus predicted values in D21_Ca model.

**Figure 7:**
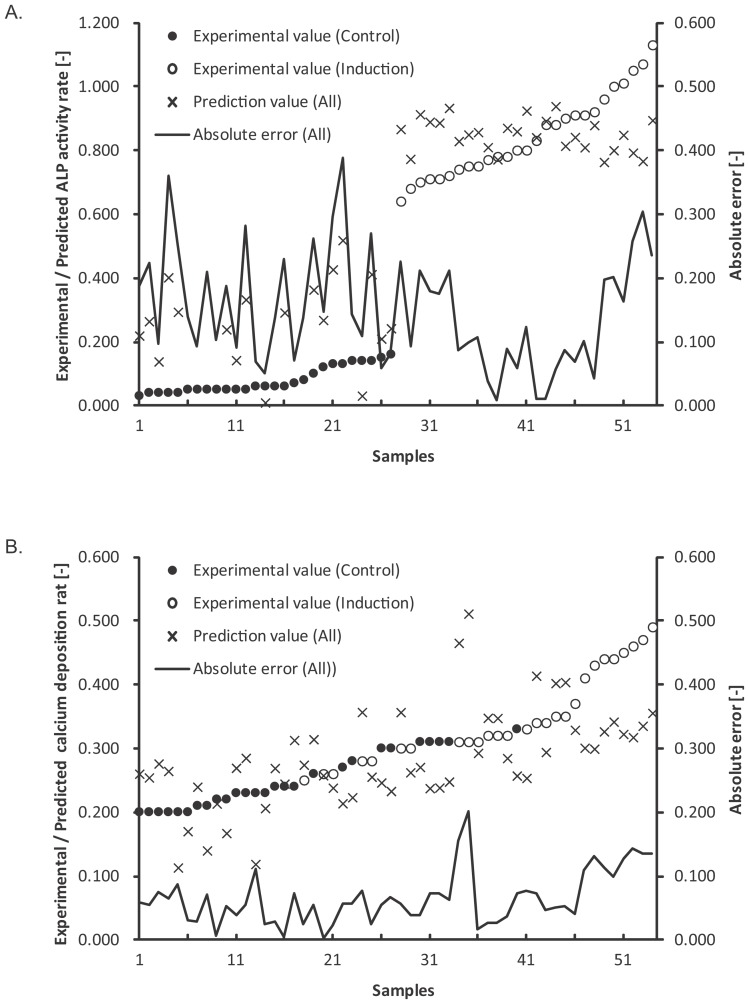
Detailed prediction results in new patient scheme. A: Prediction results and error range in the D14_ALP model. B: Prediction results and error range in the D21_Ca model. All the plotted data were rearranged in the order of experimental values.

The prediction accuracies of the D14_ALP and the D21_Ca models are shown in Table 1 and, Fig. 6 (see also Fig. 7 and Table S1 for detailed data). From both prediction results, the correlation coefficients indicated that time-course morphological features of BMSCs during differentiation correlate with the experimentally obtained osteogenic marker values. The average of absolute prediction errors indicates that each prediction model provides predictions within the error rage of ±0.151 with the D14_ALP model and ±0.065 with the D21_Ca model, respectively (Fig. 7). When the variance of all assay data, the result of manual experimental variance, is normalized as 1.0, the prediction errors between different assay measurements can be standardized as 0.194 (D14_ALP) and 0.963 (D21_Ca). This standardized error provides the interpretation that the prediction values are 5-fold stable (D14_ALP) or nearly equal (D21_Ca) compared to the human assay variances.

**Table 1:** Prediction accuracy of Ridge regression models for osteogenic differentiation status of hBMSCs.

	New patient prediction scheme		Ongoing patient prediction scheme	
	Ave. absolute prediction error* [−]	R** [−]	Ave. absolute prediction error* [−]	R** [−]
ALP activity rate prediction	0.151	0.903	**0.111**	**0.950**
Calcium deposition rate prediction	0.065	0.526	**0.037**	**0.821**

* Ave. absolute prediction error is the average of the differential between experimentally determined rate and predicted rate.

** Correlation coeffecient between experimentaly determined and predicted rate.

#### Scenario II: Ongoing patient scheme

The ongoing patient prediction scheme is designed to simulate the clinical situation of evaluating a new patient's cell quality with higher accuracy in return for additional data acquisition process (Fig. 5–B). With this scheme, a small sample of the new patient's cells should be differentiated for 14 days as a pilot culture in parallel to the expansion culture. During this pilot culture, cell images are taken to represent the new patient's cellular characteristics. The new patient's data and previous patients' historical data are combined for training the prediction model. Using image data from the next passage and from the 14 day differentiation culture, a prediction can be made prior to the cell harvest (Fig. 5–B). The advantage of this scheme is inclusion of new patient data at the cost of acquiring and inputting new patient images. Characteristics, which may be unique to the new patient, can then be incorporated into training the model with the expectation of a greater predictive value.

**Figure 8:**
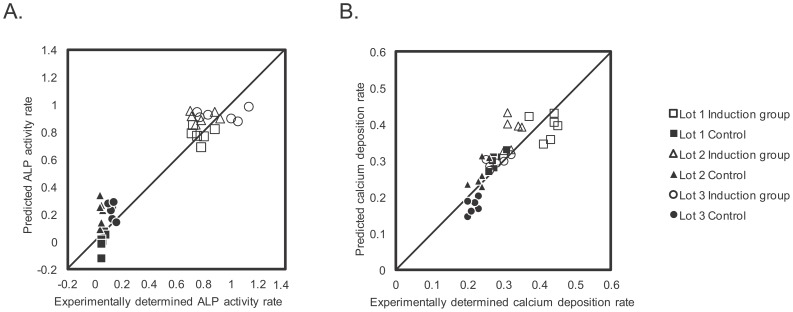
Prediction accuracies in the ongoing patient scheme. A: Scatter plot of experimentally determined values versus predicted values in D14_ALP model, B: Scatter plot of experimentally determined values versus predicted values in D21_Ca model.

**Figure 9:**
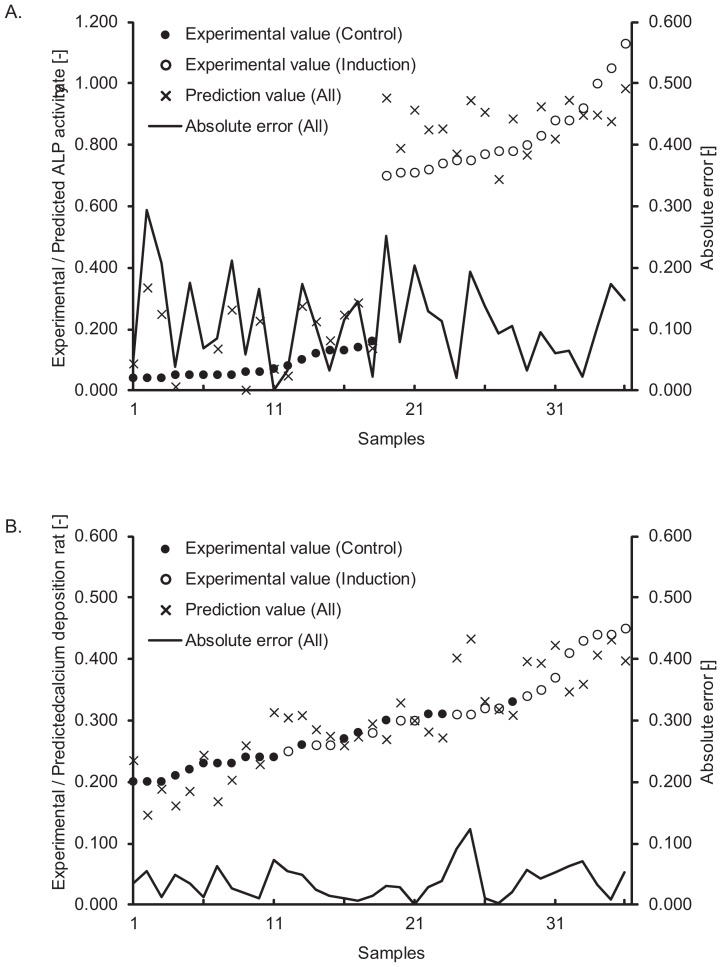
Detailed prediction results in ongoing patient scheme. A: prediction results and error range in the D14_ALP model. B: Prediction results and error range in the D21_Ca model. All the plotted data were rearranged in the order of experimental values.

The prediction results of the D14_ALP and D21_Ca models are shown in Table 1 and Fig. 8 (see also Fig. 9 and Table S2 for detailed data). These results confirm that the morphological features of hBMSCs observed during differentiation culture highly correlates with the future osteogenic potential. The error ranges were tightened to ±0.111 with D14_ALP model, and ±0.037 with D21_Ca model (Fig. 9) as compared to using only historical data to train the model. The standardization of all assay variances to 1.0 results in prediction errors of 0.110 (D14_ALP) and 0.333 (D21_Ca) respectively. Overall, these results suggest that prediction values from Scenario II morphology-based models are nearly 9-fold stable (D14_ALP) or 3-fold stable (D21_Ca) compared to the human assay variances.

When comparing the two scenarios, the prediction accuracies in both prediction models (D14_ALP and D21_Ca) greatly improved in Scenario II, the ongoing patient prediction scheme. These results indicate that incorporation of morphological characteristics from the patient's own cells is extremely important and informative for predicting an individual's BMSC osteogenic potential.

## Discussion

Although qualitative cellular morphology is used as a guide for estimating osteogenic differentiation, a quantitative relationship between cellular morphology and biochemical osteogenic markers is not well established. In the present study, we investigated the possibility of predicting osteogenic differentiation of hBMSCs from phase contrast images alone. Specifically, a machine learning algorithm was used to train 14 day cell morphology information and terminal osteogenic biochemical marker values into a model used to predict the terminal marker values from a test set of morphologic data. Our results provide evidence that using this approach can potentially automate and improve decisions, which are currently based on conventional destructive assays and qualitative microscopic assessments.

Using both ALP activity and calcium deposition rates in assessing cellular quality is important to current standard practices. Our proposed modeling schemes allow for accurate prediction of both endpoints. Of particular importance is the accurate prediction of calcium deposition, which is more closely associated with *in vivo* bone formation. For these reasons, constructing different types of prediction models to allow real-time evaluation of the same target cells with multiple aspects and add information for more careful decision making in the culture process. These attributes of an automated computational approach for assessing cellular quality support improvement of safety, efficacy and more rapid and economical scheduling decisions by physicians.

New technology allowing automated image acquisition, which can currently provide more images with greater quality and fewer biases, improves our ability to generate more predictive models based on cellular morphology. In our work, state-of-the art imaging platform (in this study BioStation CT), is the first enhancement technology which lead us to provide uniform and objective data without need for manual optimization of lighting, focusing, or other systematic errors common to manual image acquisition. Operator bias for field selection is also greatly reduced by optimization of the seeding protocol to improve cell distribution together and optimization of the number of fields to view jointly. Image processing biases, the thresholding bias to extract cells from non-cell objects recognized in the images, are also improved by a new automated threshold determination algorithm (data not shown). By preparing three different cell lots and three different passages for cell samples, we aimed to reduce biases of specific patient.

The second enhancement incorporated into this study is the incorporation of robust and continuous morphological features. In this study, it is a key that the morphological features actually used in our modeling are statistical composites (average and standard deviation) of features obtained from all cells from a given condition, which is typically comprised of approximately 4,000–40,000 cells from 15 images for each condition. Such large number of technical replicates offers robustness in each parameter, and effectively enhanced the performance of our prediction model. The continuous image acquisition with precise timing by BioStation CT allowed us to obtain both static morphological features and their dynamic changes throughout the differentiation process. Since morphological changes during osteogenic induction are time-dependent events, it is important to analyze morphological changes with precise timing.

The third enhancement provided by our work stems from the examination of two clinically plausible scenarios. Through these experiments, we found that prediction accuracies of both osteogenic potential measurements greatly increase when the model incorporates training information from early images from the same patient, which reflects individual characteristics in cell morphology. Carefully considering patient-to-patient morphology variation exposes limitations in the current practice of experience-based assessment by culture experts. As indicated in our results, use of new high-content information databases from images, where large amounts of data can be computationally organized and retrieved, is one possible approach for incorporating many aspects related to patient-to-patient variability. Constructing predictive models using historical databases and individualizing new patient predictions by incorporating each new patient's data would be a practical approach to mitigating patient variability while improving the precision of quality assessments.

Comparing the prediction errors of D14_ALP and D21_Ca models in Table 1, the D14_ALP prediction accuracy was higher. One possible explanation for the discrepancy between D14_ALP and D21_Ca predictions is the lack of morphological data during the calcium deposition period, which requires an additional week of culture following two weeks of induction culture. We plan to further investigate ways to enhance predicting this late maturation marker by accumulating more culture images to accrue a larger historical data set. However, we were surprised to discover that without the last seven days of morphology data the D21_Ca model could still predict the final calcium deposition result with reasonable accuracy. To our knowledge, no other reports have been able to accurately estimate the final calcium deposition from early images.

Previous reports have indicated that morphological parameters, similar to the ones used in this study such as flatness or polygonal rate, highly correlate with the osteogenic differentiation potential. Consistent with these reports, we looked at the contribution of each parameter to the prediction performance. In regression analysis, one can examine the effect of each parameter by examining the regression coefficients. Interestingly, among the nine parameters introduced into the regression analysis, there were few sizeable positive or negative coefficients (data not shown). This suggests that there are few dominant morphological parameters that simply correlate to the differentiation potential. Furthermore, when individual features were intentionally eliminated from the model, no significant deterioration was observed in the prediction accuracy (Table 2 and 3). These results suggest that correlation of morphological features and the osteogenic differentiation potential is so complex that there are various compensatory features. Therefore, we conclude that to gain the most robust prediction model for hBMSC osteogenic differentiation potential, all available morphological features throughout the differentiation culture should be incorporated, and biased or feeling-based morphological feature selection should be avoided.

**Table 2:** Prediction accuracy of Redge regression models with elimination of each individual features for ALP activity rate.

	New patient prediction scheme		Ongoing patient prediction scheme	
Excluded parameter	Ave. prediction error* [−]	Standardized error** [−]	Ave. prediction error* [−]	Standardized error** [−]
Breadth	0.131	0.142	0.078	0.057
Elliptical form factor	0.121	0.124	0.074	0.052
Fiber breadth	0.156	0.197	0.091	0.074
Fiber length	0.136	0.147	0.074	0.051
Hole area	0.141	0.152	0.085	0.060
Inner radius	0.138	0.146	0.088	0.065
Relative hole area	0.134	0.148	0.090	0.065
Shape factor	0.148	0.180	0.108	0.104
Total area	0.136	0.153	0.087	0.064

* Ave. prediction error is the average of the differential between experimentally determined rate and predicted rate.

** Standardized error is calculated by dividing the average of squared errors by variance of all the experimentally evaluated values.

**Table 3:** Prediction accuracy of Redge regression models with elimination of each individual features for calcium deposition rate.

	New patient prediction scheme		Ongoing patient prediction scheme	
Excluded parameter	Ave. prediction error* [−]	Standardized error** [−]	Ave. prediction error* [−]	Standardized error** [−]
Breadth	0.065	0.924	0.028	0.187
Elliptical form factor	0.066	0.972	0.026	0.166
Fiber breadth	0.062	0.870	0.026	0.158
Fiber length	0.065	0.921	0.027	0.164
Hole area	0.065	0.937	0.026	0.138
Inner radius	0.063	0.892	0.028	0.180
Relative hole area	0.060	0.769	0.025	0.146
Shape factor	0.067	0.939	0.029	0.201
Total area	0.067	0.968	0.027	0.160

* Ave. prediction error is the average of the differential between experimentally determined rate and predicted rate.

** Standardized error is calculated by dividing the average of squared errors by variance of all the experimentally evaluated values.

In this work, longitudinal morphological measurements were used as individual, unconnected features, like snapshots. However, to improve the accuracy of the D21_Ca model, we examined ways to incorporate time dependent changes of individual features. With this idea, the same morphological features were converted to change rates between sampling times, analogous to measuring the differences through snapshots. As a result, this morphological feature transformation reduced the D21_Ca model standardized error rate from 0.333 to 0.192 (data not shown). We plan to further investigate the transformation or repeated measurements into time-based trends and patterns in morphological data to improve predictive performance. Our next investigation is designed to further demonstrate applicability and robustness of our proposed method by evaluating the model's ability to characterize “cellular variances”, derived from patient diversity, culture protocol effects, and accumulating stresses throughout culture. We also plan to further expand the scope of this work to translate progress made using *in vitro* models and endpoints for morphological prediction of osteogenic potential *in vivo*.

## Materials and Methods

### Cells and cell culture

Human bone-marrow derived mesenchymal stem cells (hBMSCs) (Lonza Walkersville, Inc., Maryland, U.S.A.) were subcultured (passaged) in Dulbecco's modified Eagles' medium (DMEM) containing 10% fetal bovine serum (FBS) (Life Technologies Japan Ltd., Tokyo, Japan). Three lots of hBMSCs were designated as Lot 1 (strain number 15000-1, unknown race, Male, 19-year-old), Lot 2 (strain number 17174, Oriental, Male, 20-year-old), and Lot 3 (strain number 11533, Black, Male, 22-year-old), respectively. Lot 1 and 2 were cultured to passages 3, 4, and 5, and Lot 3 was cultured to passages 6, 7, and 8, and cryopreserved for the start of the image acquisition experiment. Cryopreserved cells were seeded at a density of 1.0×10^4^ cells/well in 12-well plate (Greiner Bio-One., Frickenhausen, Germany), and the cell-seeding day was designated as day 0 in the image acquisition experiment.

Fig. 1 illustrates the experimental scheme for hBMSC osteogenic differentiation culture. From day 0 to 3, cells were cultured in 10% FBS-containing α-modified Eagle's medium (αMEM) (Sigma-Aldrich Co., St. Louis, MO, U.S.A.). From day 4 to 18, cells were divided into two groups: (1) Osteogenic induction group (Induction, N = 6) and (2) Non-induction group (Control, N = 6). For the induction group, the medium was switched to induction medium consisting of 10% FBS-containing αMEM medium supplemented with 10 nM dexamethasone (Sigma-Aldrich Co.), 100 mM ascorbic acid (Wako Pure Chemical Industries, Ltd., Osaka, Japan), and 10 mM glycerol 2-phosphate sodium salt hydrate (Sigma-Aldrich Co.). For the non-induction group, supplements were not added to the 10% FBS-containing αMEM medium. The appropriate medium was changed at day 7 and day 13. For half of the samples in each experimental group (n = 3), alkaline phosphatase (ALP) activity were quantified on day 18. The remaining samples continued culture until day 25, and calcium deposition was quantified on day 25.

### Image acquisition

**Figure 10:**
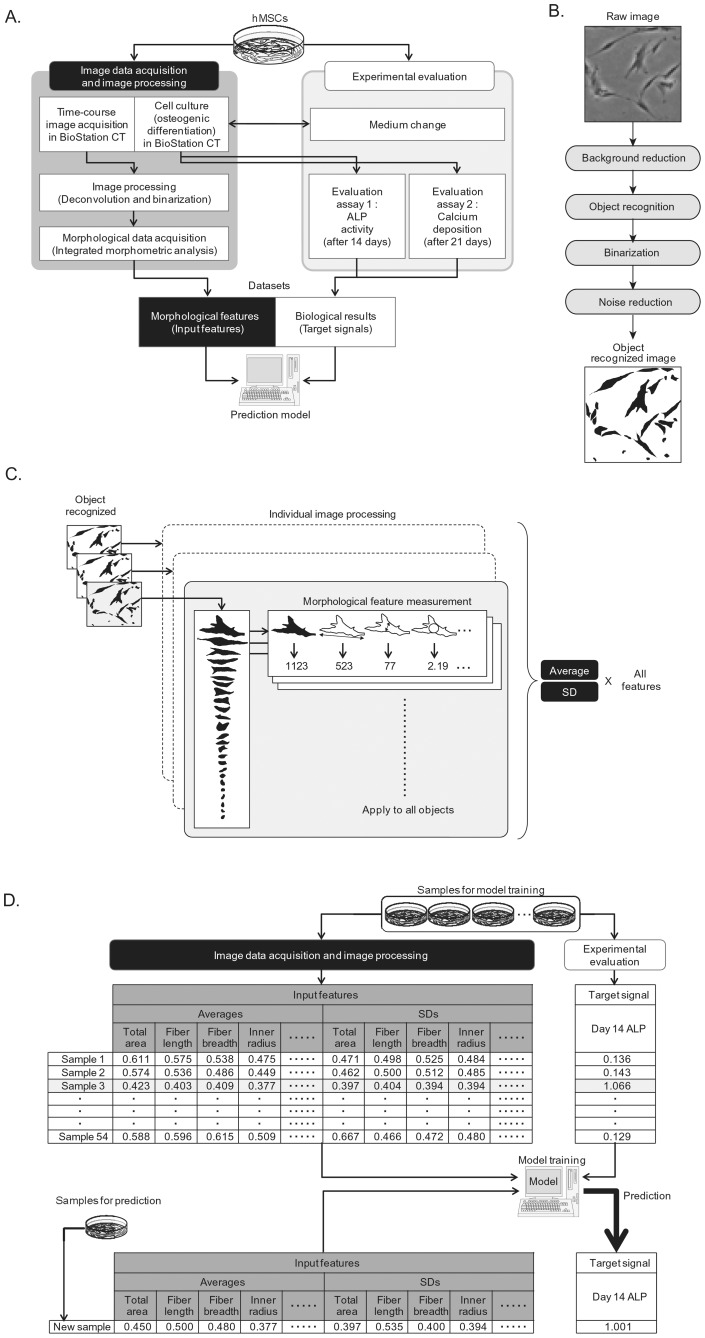
A: Schematic illustration of cell image processing. The raw images were first pre-processed by background reduction processing by deconvolution and open-close filters. Then, images were binarized by the optimized threshold. The noisy objects were eliminated by particle deletion filter. B: Schematic illustration of cell morphology measurements and data processing. In all object recognized images, all existing objects were measured for the 9 morphological features. Since 1 condition was designed to consist of 3 wells ×5 view fields, all the corresponding object measurement results were processed as a same sample. The average and standard deviation within one sample of all morphological features at each time point were used as the input features for modeling. C: Schematic illustration of prediction model construction. Prediction of differentiation potential consisted of two steps. First, two types of prediction models (D14_ALP model or D21_Ca model) were constructed with the set of image data and experimental evaluation. Second, the values of D14_ALP or the D21_Ca were predicted from the input features of the sample targeted for prediction. The predicted biological rates are compared to the experimentally-determined results to evaluate the accuracy of prediction model.

Figs. 1 and 10-A illustrates the image acquisition scheme during hBMSC osteogenic differentiation culture. From day 0 to day 13 (14 days), phase contrast microscopic images of hBMSCs were obtained using the BioStation CT (Nikon Corporation, Tokyo, Japan). BioStation CT is an automatic cell maintenance system, which maintains a stable incubation environment (37°C, 5% CO_2,_ 100% humidity) with scheduled automatic image acquisition. The number of view fields was optimized to five, which provides the least error for estimating the correct cell seeding for each well. Five view fields (center position and four positions 2.2 mm from the center) of phase contrast images were acquired from each well with fully automatic focusing. The phase contrast images had the least noise and background when using the halo-reduction lens. Image acquisition timing was set to every 8 hours from day 4 to 18 (magnification  = 10x). Time points are designated as time 0 to 38, indicating each of the 8 hour imaging intervals. Data at time 0 was omitted, since the cells were not fully settled. Data at time 7 and time 26 were also omitted, since it was concurrent with medium changes for each well plate, resulting in 36 time-points as a total.

### Quantification of ALP activity

Quantitative ALP activity assays were performed as previously described [9]. After 18 days of culture, cell number was measured using a cell counting kit-8 (WST-8®; Dojindo Laboratories, Kumamoto, Japan), and ALP activities were measured with a p-nitrophenyl phosphate solution (Lab Assay ALP®; Wako Pure Chemical Industries, Ltd.). Briefly, for cell count, 100 µl of WST-8 was added to each well containing 1 mL of fresh medium, incubated for 1 hour, and absorbance was read at 450 nm. After WST-8 analysis, each well was washed twice with phosphate buffered saline (PBS) and 800 µL of p-nitrophenyl phosphate solution was added to each well. After 10 min of incubation at 37°C, the conversion to p-nitrophenol was stopped with 800 µl of 3N NaOH and the absorbance of p-nitrophenol was measured at 405 nm. Alkaline phosphatase-specific activity is expressed as p-nitrophenol absorbance (OD; 405 nm)/WST-8 absorbance (OD; 450 nm).

### Calcium deposition quantification

After 25 days of culture, cells were fixed with 70% ethanol for 1 hour, washed, and stained for 10 min with 40 mM alizarin red S solution (pH: 4.2). After washing with PBS, plates were incubated with 10% cethylphridinium chloride for 15 min. Thereafter, supernatants were collected from each well and the absorption of each supernatant was measured at 405 nm to determine the amount of calcium deposition.

### Cell image processing

All images (.bmp files) were processed by MetaMorph (Molecular device, CA, U.S.A) with the original combination of image-processing filter sets (Fig. 10–A). Briefly, the raw images were pre-processed by open-close filters and binarized by the optimized threshold. All image data was pre-processed using the same brightness threshold, which was optimized by 20 randomly picked image samples. This pre-processing step minimized error between the manually determined image cell number and the number of objects determined after pre-processing. After binarization, all individual objects in each image, consisting of cells and noise (non-cell objects), were measured by the integrated morphometric analysis function to measure morphological features (9 morphological features are: Breadth, Elliptical form factor, Fiber breadth, Fiber length, Hole area, Inner radius, Relative hole area, Shape factor, Total) (Fig. 10–B). The morphological features were carefully selected with the MetaMorph measurement function by logical selection. Features related to color and brightness were excluded first. Second, independent features were selected by hierarchical clustering and highly correlated features (R>0.85) were excluded. From the data consisting of object ID and its standardized 9 morphological features (average  = 0, standard deviation  = 1), the noise data (non-cell objects) was automatically cleansed by the original noise-reduction algorithm prior to the analysis (patent pending). From the pre-processed data, average (AVE) and standard deviation (SD) from each of the 9 morphological features was calculated from each of the cell objects covering five view fields from the same well, and used as the 18 inputs (9 features with AVE and SD) for each sample to be used in further analysis (Fig. 10–C). The morphological features and cell number (AVE and SD for 19th and 20th feature) from each well were then tagged with the target signals, which are experimentally determined values, resulting in 54 samples ( = 3 lots ×2 induction conditions ×3 passages ×3 wells) tagged with ALP values, and 54 samples tagged with calcium deposition values. This process links the “result” (biological measurement) with the “indication” (image-derived morphological feature), to derive a dataset for further modeling (Fig. 10–D).

### Construction and evaluation of prediction model

Prediction of differentiation potential consists of two steps (Fig. 10–D): one is the construction of a prediction model, and the other is the evaluation of the constructed model. Using Ridge regression, two types of prediction models were constructed: (1) D14_ALP model, and (2) D21_Ca model. For the new patient scheme, prediction models were trained with 36 samples from 2 lots, and 18 samples from the remaining single lot were predicted. For the ongoing patient scheme, prediction models were trained with 42 samples from 2 lots of 3 passages plus the samples from new lot of 1 or 2 passages were used for training, and 12 samples from the remaining 1 lot were predicted (Fig. 5). The detailed modeling process is described in a previous report (See Section 3 in [18] for details of the Ridge regression method). The performance of each of the models and datasets were evaluated by the average accuracy resulting from leave-one-out cross validation.

For the evaluation of our proposed scheme, two evaluation indices are introduced in our work. One index is the correlation coefficient (R) of actual assay values and prediction values, which evaluates the prediction accuracy and its data coverage. The higher R increases, the more the model is capable of predicting “differentiation marker values” with small error rate. The other index of evaluation that we introduced is the average of absolute error. This value is calculated by obtaining absolute values of (experimentally determined value minus the predicted value). To compare these errors, we standardized these errors by dividing the variance of total experimentally determined values in one assay.

## Supporting Information

Table S1 * Exp. determined rate is the abbreviation of the experimentally determined rate.
(XLS)

Table S2 * Exp. determined rate is the abbreviation of the experimentally determined rate.
(XLSX)

## References

[1] Wu Y, Chen L, Scott PG, Tredget EE (2007) Mesenchymal stem cells enhance wound healing through differentiation and angiogenesis. Stem Cells 25: 2648–2659. 17615264 10.1634/stemcells.2007-0226

[2] Chen L, Tredget EE, Wu PY, Wu Y (2008) Paracrine factors of mesenchymal stem cells recruit macrophages and endothelial lineage cells and enhance wound healing. PLoS ONE 3: e1886. 18382669 10.1371/journal.pone.0001886 PMC2270908

[3] Barry FP, Murphy JM (2004) Mesenchymal stem cells: clinical applications and biological characterization. Int J Biochem Cell Biol 36: 568–584. 15010324 10.1016/j.biocel.2003.11.001

[4] Hayashi O, Katsube Y, Hirose M, Ohgushi H, Ito H (2008) Comparison of osteogenic ability of rat mesenchymal stem cells from bone marrow, periosteum, and adipose tissue. Calcif Tissue Int 82: 238–247. 18305886 10.1007/s00223-008-9112-y

[5] Mizuno D, Kagami H, Mizuno H, Mase J, Usami K, et al (2008) Bone regeneration of dental implant dehiscence defects using a cultured periosteum membrane. Clin Oral Implants Res 19: 289–294. 18081869 10.1111/j.1600-0501.2007.01452.x

[6] Olivo C, Alblas J, Verweij V, van Zonneveld AJ, Dhert WJ, et al (2008) In vivo bioluminescence imaging study to monitor ectopic bone formation by luciferase gene marked mesenchymal stem cells. J Orthop Res 26: 901–909. 18271011 10.1002/jor.20582

[7] Kagami H, Agata H, Tojo A (2011) Bone marrow stromal cells (bone marrow-derived multipotent mesenchymal stromal cells) for bone tissue engineering: Basic science to clinical translation. Int J Biochem Cell Biol. 43: 286–289. 10.1016/j.biocel.2010.12.006 21147252

[8] Dennis JE, Esterly K, Awadallah A, Parrish CR, Poynter GM, et al (2007) Clinical-scale expansion of a mixed population of bone-marrow-derived stem and progenitor cells for potential use in bone-tissue regeneration. Stem Cells 25: 2575–2582. 17585167 10.1634/stemcells.2007-0204

[9] Agata H, Asahina I, Yamazaki Y, Uchida M, Shinohara Y, et al (2007) Effective bone engineering with periosteum-derived cells. J Dent Res 86: 79–83. 17189468 10.1177/154405910708600113

[10] Platt MO, Wilder CL, Wells A, Griffith LG, Lauffenburger DA (2009) Multipathway kinase signatures of multipotent stromal cells are predictive for osteogenic differentiation: tissue-specific stem cells. Stem Cells. 27: 2804–2814. 10.1002/stem.215 PMC2976759 19750537

[11] Kelly DJ, Jacobs CR (2010) The role of mechanical signals in regulating chondrogenesis and osteogenesis of mesenchymal stem cells. Birth Defects Res C Embryo Today. 90: 75–85. 10.1002/bdrc.20173 20301221

[12] Kino-Oka M, Maeda Y, Sato Y, Maruyama N, Takezawa Y, et al (2009) Morphological evaluation of chondrogenic potency in passaged cell populations. J Biosci Bioeng (107): 544–551. 10.1016/j.jbiosc.2008.12.018 19393556

[13] Carpenter AE, Jones TR, Lamprecht MR, Clarke C, Kang IH, et al (2006) CellProfiler: image analysis software for identifying and quantifying cell phenotypes. Genome Biol. 7: R100. 10.1186/gb-2006-7-10-r100 PMC1794559 17076895

[14] Misselwitz B, Strittmatter G, Periaswamy B, Schlumberger MC, Rout S, et al (2010) Enhanced CellClassifier: a multi-class classification tool for microscopy images. BMC Bioinformatics. 11: 30. 10.1186/1471-2105-11-30 PMC2821321 20074370

[15] Harder N, Mora-Bermudez F, Godinez WJ, Wunsche A, Eils R, et al (2009) Automatic analysis of dividing cells in live cell movies to detect mitotic delays and correlate phenotypes in time. Genome Res. 19: 2113–2124. 10.1101/gr.092494.109 PMC2775592 19797680

[16] Jones TR, Carpenter AE, Lamprecht MR, Moffat J, Silver SJ, et al (2009) Scoring diverse cellular morphologies in image-based screens with iterative feedback and machine learning. Proc. Natl. Acad. Sci. U. S. A. 106: 1826–1831. 10.1073/pnas.0808843106 PMC2634799 19188593

[17] Hoerl AE, Kennard R (1970) Ridge regression: biased estimation for nonorthogonal problems. Technometrics 12: 55–67.

[18] Hastie T (2009) The Elements of Statistical Learning: Data Mining, Inference, and Prediction. Springer-Verlag. 43.

